# The impact of anticoagulant-related bleeding on quality of life: Development of a novel measure based on perspectives from older adults

**DOI:** 10.1371/journal.pone.0316796

**Published:** 2025-01-29

**Authors:** Anna L. Parks, Stacey L. Slager, Amy M. Cizik, Margaret C. Fang, Mark A. Supiano, Patti P. Katz, Daniel M. Witt

**Affiliations:** 1 Division of Hematology & Hematologic Malignancies, University of Utah, Salt Lake City, Utah, United States of America; 2 Conflict of Interest Office, University of Utah, Salt Lake City, Utah, United States of America; 3 Department of Orthopedics, University of Utah, Salt Lake City, Utah, United States of America; 4 Division of Hospital Medicine, University of California, San Francisco, California, United States of America; 5 Division of Geriatrics, University of Utah, Salt Lake City, Utah, United States of America; 6 Division of Rheumatology, University of California, San Francisco, California, United States of America; 7 Department of Pharmacotherapy, University of Utah, Salt Lake City, Utah, United States of America; Bursa Ali Osman Sonmez Oncology Hospital, TÜRKIYE

## Abstract

**Background:**

Venous thromboembolism (VTE) and atrial fibrillation (AF) disproportionately affect older adults, who are at increased risk of bleeding from treatment with anticoagulant therapy. The impact of bleeding on older adults’ quality of life (QoL) is poorly understood due to the lack of a validated measure of their experience. This study’s purpose is to describe the first evidence-based steps in developing a new condition-specific patient-reported outcome measure (PROM) for the effect of anticoagulant-related bleeding on older adults’ QoL.

**Methods:**

Adults aged 65 years and older with VTE or AF at the University of Utah who were eligible for anticoagulation were recruited. We purposely sampled by age, sex, race/ethnicity, diagnosis, bleeding history and anticoagulant. In accordance with evidence-based guidelines for PROM development, focus groups were conducted to refine a hypothesized conceptual model of bleeding-related QoL and analyzed according to hybrid inductive and deductive thematic analysis methods. Focus group results were used to write a draft PROM. Semi-structured qualitative interviews were conducted to revise items on the draft PROM and were analyzed using thematic analysis to generate a final bleeding-related QoL PROM.

**Results:**

Twenty individuals participated in interviews, and nine in two focus groups. Median age was 81 (range 69–95), 52% were women and 69% were White (10% Asian, 10% Hispanic, 7% Black, 3% Native Hawaiian/Pacific Islander). Through inductive and deductive thematic analysis of focus groups, we identified 19 themes (e.g., specific bleeding symptoms, adaptations in relationships, fatigue) that fell into 5 domains: 1) bleeding symptoms, 2) healthcare experiences, 3) social function, 4) emotional function, 5) physical function. These 19 themes and 5 domains formed the basis of the draft PROM. We conducted semi-structured cognitive interviews, and we performed thematic analysis to revise the drafted measure for clarity and relevance. The data resulted in a new PROM for bleeding-related QoL in older adults.

**Conclusion:**

Employing evidence-based methods for PROM development, we found that bleeding can have a marked impact on everyday activities, emotional wellbeing and interpersonal relations for older adults. We incorporated these distinct aspects of wellbeing and function into a novel PROM for bleeding-related QoL that can inform clinical care and research after subsequent psychometric validation.

## Introduction

Anticoagulant therapy is highly effective at preventing thromboembolic stroke in atrial fibrillation (AF) and treating venous thromboembolism (VTE), but bleeding risk associated with treatment increases considerably with age. Each advancing decade in age results in a 30% increase in the relative risk of bleeding on anticoagulation, and anticoagulant-related bleeding is the most common cause of emergency room visits among older adults [1, 2]. Thus, as the population of older adults taking anticoagulants grows, anticoagulant-related bleeding represents a mounting public health challenge.

Despite its increasing prevalence, existing anticoagulant-related bleeding instruments do not adequately measure the impact of bleeding on older adults’ quality of life (QoL). Currently, bleeding complications are classified as major bleeding, which are large-volume hemorrhages into a critical site or that require transfusion, or clinically relevant non-major bleeding, which are less severe hemorrhages that still require in-person medical attention [3–5] These research definitions of bleeding events do not capture small-volume bleeds (so-called “nuisance bleeding”) and do not address bleeding’s effects on patient-centered outcomes like independence and physical function and thus may not represent older adults’ experiences. The two most commonly used existing patient-reported outcome measures (PROMs) are treatment-specific: the Duke Anticoagulation Satisfaction Scale (DASS) and the Anti-Clot Treatment Scale (ACTS) [6, 7]. The DASS contains 25 questions that focus on the limitations, hassles, burdens, confidence, reassurance, and satisfaction of treatment with anticoagulant medications. The ACTS consists of 12 items focused on anticoagulant treatment burdens and 3 items focused on treatment benefits. Notably, the DASS and the ACTS questions measure treatment satisfaction with anticoagulant medications rather than anticoagulant-related bleeding and its effect on wellbeing and function. Moreover, neither measure was developed or validated in older adults. The unique complexities of older adults, including frailty and multimorbidity, may influence how bleeding affects QoL. Moreover, bleeding in older adults may be multifactorial due to aging-related risk factors or anticoagulant effect, and these nuances are not captured by existing measures. Efforts to align clinical care with older adults’ values are hindered by a lack of a validated measure of older adults’ real-world experience of bleeding. To respond to this gap, the purpose of this paper is to detail the first stages of development of a new condition-specific PROM for the effect of anticoagulant-related bleeding on older adults’ QoL. First, we detail development of a new conceptual model of bleeding-related QoL for older adults using focus groups. Secondly, we describe pilot testing of a novel PROM using semi-structured qualitative interviews and expert review.

## Methods

### Overview of study

**Fig 1:**
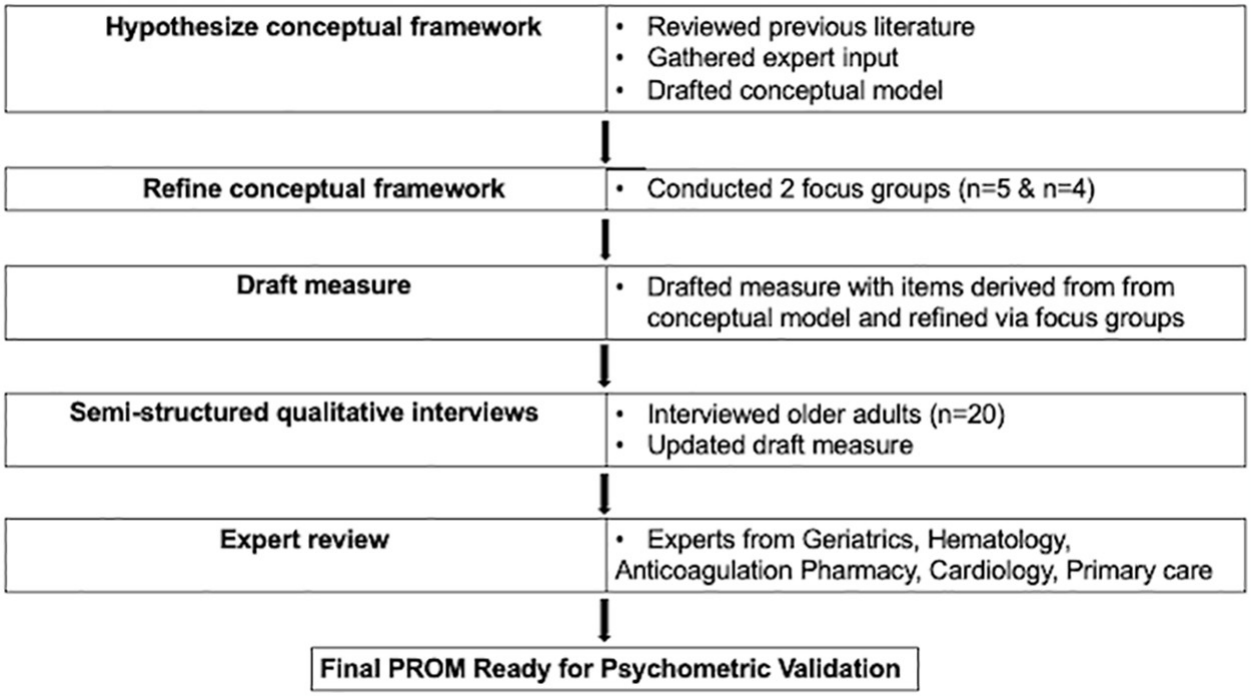
Summary of steps to develop a novel patient-reported outcome measure for bleeding-related quality of life in older adults.

As we depict in Fig 1, here we report key first steps in development of a novel, condition-specific PROM as recommended by the Food and Drug Administration (FDA) and the COSMIN (COnsensus-based Standards for the selection of health Measurement INstruments) group [8, 9]. First, we developed a conceptual model for the pilot questionnaire based on a broad literature review, previously validated instruments, and expert review. We next conducted focus groups to refine our hypothesized model. After revising the conceptual model based on focus group data, we drafted a pilot measure with an item pool derived from the refined conceptual model’s themes and domains. We then conduced semi-structured qualitative interviews with older adults to revise the draft measure. Multi-disciplinary experts then reviewed the resulting draft measure to develop a PROM that is ready for psychometric validation.

### Eligibility and recruitment

For both focus groups (to refine the conceptual model) and interviews (to evaluate the pilot measure), we recruited participants aged 65 years or older (a standard definition of older adults), with a diagnosis of AF or VTE [10, 11]. To avoid selection bias, since older adults who are prescribed anticoagulants are healthier and thus may have different bleeding rates and QoL than those who are not, we included those eligible for but not taking anticoagulant medications. Participants were recruited from the University of Utah Hematology, Cardiology, Geriatrics, Primary Care and Thrombosis clinics between October 1, 2021 and March 31, 2022. The University of Utah is the only academic medical system in the Intermountain West, serving nearly 10 million patients living in Utah, Idaho, Wyoming, Montana, Nevada and western Colorado. Potential subjects were identified in one of two ways. First, the principal investigator examined clinic schedules and reviewed the charts of patients who were age 65 years and older with a listed diagnosis of AF or VTE. Additionally, to capture subjects who might not have had a recent clinic appointment, the principal investigator reviewed pharmacy records for adults over age 65 dispensed the vitamin K antagonist warfarin or direct oral anticoagulants from January and February 2022. For both recruitment methods, we performed additional chart review to confirm a diagnosis of AF or VTE and determine eligibility for anticoagulation based on international society guidelines for AF stroke prevention and VTE treatment [12, 13].

To refine the conceptual model, the FDA and COSMIN guidelines recommend conducting at least one focus group, so we estimated one to two focus groups to achieve saturation according to this guidance [8, 9, 14–17]. To evaluate the pilot measure using interviews, we estimated an a priori sample size of 20 to 30, which is an evidence-based recommended sample size to achieve data saturation when developing novel PRO instruments [18]. Potential subjects were first sent a recruitment letter followed by a telephone call to provide additional information, answer questions about the study, and schedule either a focus group or interview according to their preference. We purposefully sampled by race, gender and ethnicity to recruit a population representative of older adults in the surrounding area (Salt Lake County), which is 55% female, 81% Caucasian/White, 2% Black/African American, 1% American Indian/Alaska Native, 4% Asian, 2% Native American/Pacific Islander, 17% Hispanic/Latino [19]. We also purposefully sampled by additional factors that affect bleeding risk and quality of life, including: 1) diagnosis of AF and VTE, the two most common indications for anticoagulation (we aimed to recruit at least half of participants with VTE); 2) history of bleeding (we aimed to recruit 10% with a history of major or clinically relevant non-major bleeding by ISTH definitions described above), 3) and type of anticoagulant (we aimed to recruit 10% taking warfarin and 90% newer direct oral anticoagulants [DOACs]) [14]. We excluded those who were unable to read and speak English, were too ill to complete the focus group or interview or who were eligible for anticoagulants for any reason other than AF or VTE.

### Procedures for focus groups and semi-structured qualitative interviews

Participants were offered a choice to participate in either focus groups or semi-structured interviews, which allowed for different participant preferences and for interactions with other participants that could yield new insights. According to evidence-based practices, both focus group and interview guides were developed based on clinical knowledge and literature review by experts in anticoagulation, geriatrics, psychometrics and instrument development and qualitative methods (see S1 File for guides) [8, 9, 15, 16]. The principal investigator (A.P.) and a research assistant (S.S.), both of whom were trained in discussion moderation and interview technique, conducted focus groups and interviews. All sessions were observed by at least two investigators. This study was performed in line with the principles of the Declaration of Helsinki. Approval was granted by the Ethics Committee of the University of Utah (IRB_00146636). The institutional review board approved all study procedures, including a waiver of documentation of consent. Study participants were given an electronic copy of the study consent form prior to interviews or focus groups. The consent form includes a statement that by participating in an interview or focus group, subjects gave their consent to participate in this study and to be audio-recorded. At the beginning of each focus group or qualitative interview, this consent form and statement was reviewed with each participant, and all participants gave informed verbal consent. Interviews and focus group sessions lasted approximately one hour. Sessions were audiotaped with participants’ permission and transcribed verbatim. Participants were provided with $25 as compensation for their time. Repeat interviews or focus groups were not performed.

For focus groups to refine the conceptual model, the moderator guide presented a hypothesized conceptual model for the effect of bleeding on QoL [14, 17, 20]. Moderators asked for participant feedback on the model. Participants were also encouraged to freely express their thoughts on bleeding’s effects on their health in all dimensions. Two focus groups (n = 4 and n = 5) were conducted between November 2021 and January 2022. Because of the ongoing COVID-19 pandemic, focus groups were conducted remotely using video technology.

For semi-structured qualitative interviews of the drafted items, respondents completed the pilot version of the novel measure. The interview guide included scripted and unscripted prompts following each item [14, 17, 20]. These prompts asked participants to describe how they processed and interpreted each question. They asked about the pilot measure’s clarity of phrasing, any redundancy, any particularly important items, and any unimportant items [15, 16]. Twenty semi-structured qualitative interviews were conducted between February and June 2022. We conducted interviews via telephone.

### Data analysis

We adhered to Consolidated Criteria for Reporting Qualitative Studies (COREQ) guidance for qualitative data reporting (S1 Checklist), as well as FDA and COSMIN guidance for PROM development [8, 9, 21]. Transcribed qualitative data from the focus groups (to refine the conceptual model) and semi-structured qualitative interviews (to evaluate the pilot measure), were coded and analyzed concurrently. Transcripts were not returned to participants for this study. To minimize bias, two investigators trained in qualitative analysis (A.P. and S.S.) independently read and coded transcripts from both the focus groups and semi-structured interview phases; these same investigators conducted the focus groups and interviews. Afterward, investigators met to discuss and resolve differences in coding and to discuss reflexivity, or how the researchers’ identities and values might impact our findings. Discussions focused on researchers’ own experiences with the healthcare system or with older adults. We then re-reviewed interview transcripts to ensure that the coding scheme explored participants’ experiences and reflected their experiences rather than our own. The datasets generated and analyzed during the current study contain potentially sensitive information and are not publicly available due to the potential for loss of privacy as required by the research ethics committee. but are available from the corresponding author and research ethics committee (irb@hsc.utah.edu) on reasonable request.

Focus group data to refine the conceptual model were analyzed using hybrid inductive and deductive thematic analysis methods [14–17, 22–24]. This refined model was subsequently confirmed based on review by investigators with broad areas of clinical and methodological expertise (Geriatrics, Hospital Medicine, psychometrics and qualitative methods, Thrombosis and Anticoagulation Interview data evaluating the pilot measure were analyzed by thematic analysis [14–17, 22–24].

Saturation analyses are recommended by the FDA and COSMIN to confirm the sample size is adequate to fully explore the concepts of interest [8, 9, 25, 26]. To do so, after the first focus group and after every 3 interviews, researchers established when participants first spontaneously discussed each concept. For cognitive interviews, in addition to assessing saturation, researchers also reviewed the transcript and discussed whether PROM items should be modified, added, or removed. We defined saturation as the point at which no new relevant or important information emerges with additional data collection [25, 26]. For cognitive interviews, after 13 interviews, no new domains or themes emerged, and no item revision was required (S1 Table). The following 7 interviews confirmed that the symptom and QoL impact of bleeding had been fully explored and no further interviews were required.

## Results

A total of 29 individuals participated, nine in two focus groups (n = 5 and n = 4) and 20 in semi-structured qualitative interviews [26]. Five participants who were approached declined to participate in the study. As outlined in Table 1, we largely achieved our purposeful sampling goals, with the exception of underlying diagnosis. Combining participants in focus groups and interviews, median age was 81 years (range 69–95), 52% were women. Sixty-nine percent were White, 10% Asian, 10% Hispanic, 7% Black, 3% Native Hawaiian/Pacific Islander. Eight (28%) had VTE (versus our goal of 50%), and 21 (72%) had AF, with mean CHA_2_DS_2_-VASc score for annual stroke risk of three (standard deviation 1). Twenty-three (79%) were prescribed long-term anticoagulants, including 7 (24%) prescribed the vitamin K antagonist warfarin, and 16 (55%) prescribed direct-acting oral anticoagulants (12 [41%] apixaban and 4 [14%] rivaroxaban), while 6 (21%) were eligible for but not taking anticoagulants. Eight (28%) had a history of major or clinically relevant non-major bleeding. Two (7%) were within the first 3 months of taking an anticoagulant, while the remainder had been anticoagulated for 1 or more years.

**Table 1:** Demographic and clinical characteristics of focus group and semi-structured qualitative interview participants.

Characteristic	Focus group participants (N = 9)	Interview participants (N = 20)	All participants (N = 29)
Age, mean; SD	79 (69–91)	81 (69–95)	80 (69–95)
Female sex, n (%)	5 (56)	10 (50)	15 (52)
Race & ethnicity, n (%)			
Asian	1 (11)	2 (10)	3 (10)
Black/African-American	0 (0)	2 (10)	2 (7)
Hispanic	1 (11)	2 (10)	3 (10)
White	7 (77)	13 (65)	20 (69)
Other	0 (0)	1 (3)	1 (3)
Diagnosis, n (%)			
Venous thromboembolism	3 (33)	5 (25)	8 (28)
Atrial fibrillation	6 (67)	15 (75)	21 (72)
CHA_2_DS_2_, mean (SD)	3 (1)	3 (1)	3 (1)
Anticoagulant, n (%)	9 (100)	14 (70)	23 (79)
Warfarin, n (%)	3 (33)	4 (20)	7 (24)
Direct oral anticoagulants, n (%)	6 (67)	10 (50)	16 (55)
Apixaban	4 (44)	8 (40)	12 (41)
Rivaroxaban	2 (22)	2 (10)	4 (14)
History of bleeding, n (%)	0 (0)	8 (40)	8 (28)
Duration of therapy, n (%)			
Less than 3 months	1 (11)	1 (5)	2 (7)
More than one year	8 (89)	13 (65)	21 (72)

### Results of focus groups to refine the conceptual model

**Fig 2:**
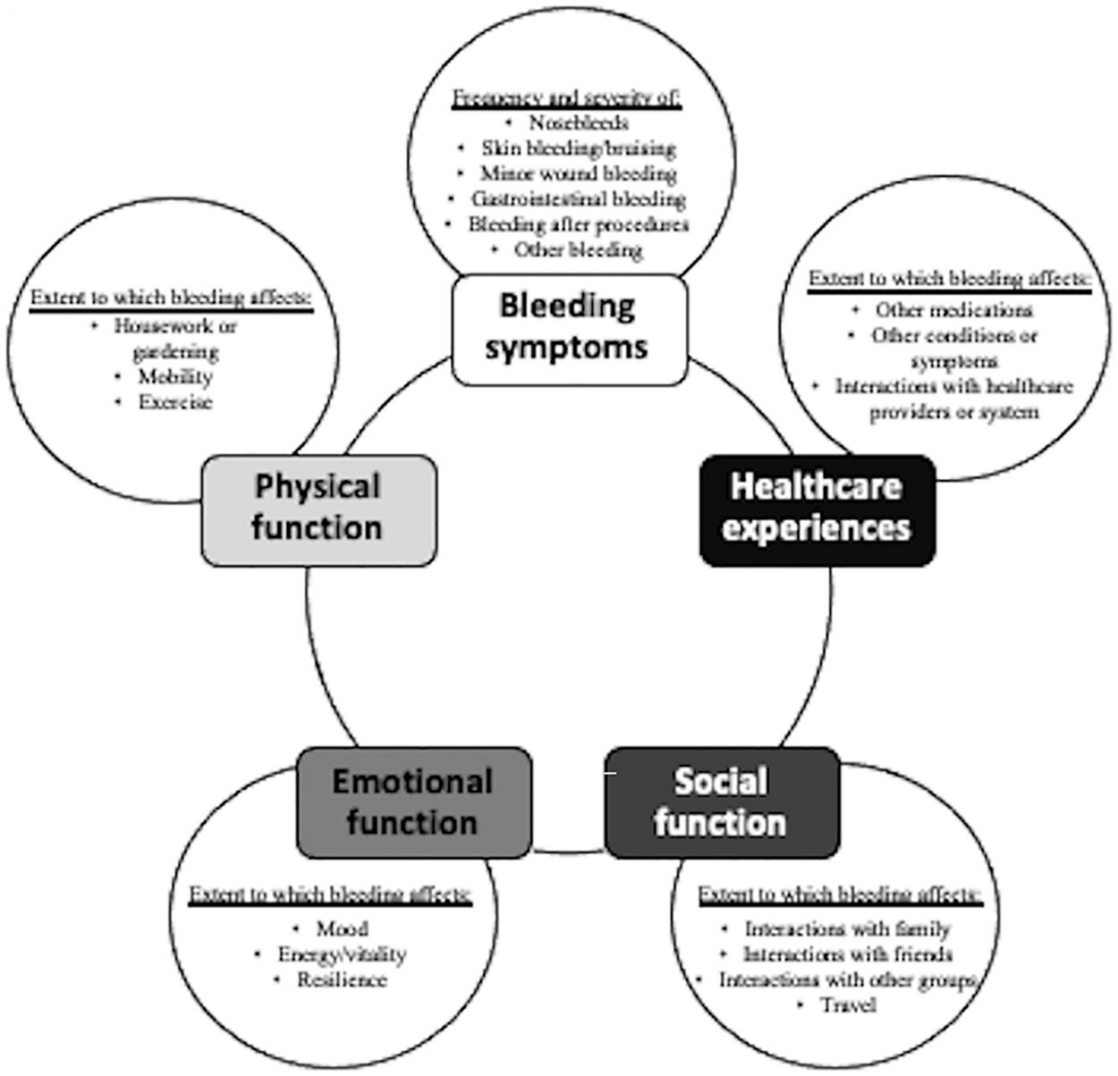
Revised conceptual model of 5 domains and 19 themes of quality of life affected by bleeding based on focus group data.

Our hypothesized conceptual model contained four domains (bleeding symptoms, physical function, emotional function, and social function). Through focus groups, nineteen dimensions of functioning and wellbeing were identified after analysis (Fig 1): specific bleeding symptoms (nosebleeds, skin bleeding/bruising, minor wound bleeding, gastrointestinal bleeding, bleeding after procedures, other bleeding); bleeding’s interaction with other medications, medical conditions or symptoms; bleeding’s effect on interactions with family, friends, other groups, and travel; bleeding’s effect on mood, energy/vitality, and sources of resiliency; and bleeding’s effect on housework or gardening, mobility and exercise. Further analysis categorized the dimensions into domains, including addition of one domain (healthcare experiences), for a total of five major domains in our final conceptual model: 1) bleeding symptoms, 2) healthcare experiences, 3) social function, 4) emotional function, and 5) physical function (Fig 2). All nine (100%) of participants reported that these domains were comprehensive of their bleeding-related QoL, easy to understand, and would be relevant to gaining a better understanding of older adults’ experiences.

### Bleeding symptoms

All (100%) participants described the importance of bleeding symptoms, but manifestations and impact on QoL varied across participants. Some described being very bothered by bleeding symptoms, remarking: “To talk about a blood draw just brings me to my knees. They eventually get the blood they need, but they have to poke me many times, and I end up with a lot of bruises where they’ve tried to go,” (female, 81 years old, on apixaban for atrial fibrillation) and, “I had a hard fall and had bruising on my thigh that took months to go away.” (female, 86 years old, on apixaban for atrial fibrillation) Four (44%) described bleeding symptoms as a minor annoyance, saying, “I garden a lot, and I have boysenberries that I have to prune. In the spring you can always tell because I have all these spots on my arms from where I’m hitting the canes from the bruising,” (female, 81 years old, on apixaban for atrial fibrillation) and “I’m subject to bleeding only in my sinuses—minor, not debilitating but unattractive. It’s not very nice, but it’s a fact.” (male, 86 years old, on rivaroxaban for venous thromboembolism).

### Healthcare experiences

Eight of nine (89%) participants spontaneously brought up how bleeding affected their interactions with healthcare providers and the healthcare system, leading us to add this to the conceptual model as a fifth domain. Two (22%) participants noted being monitored closely for bleeding during their care, remarking for instance, “My provider tells me to be careful almost every time we meet, and I try to do that,” (male, 86 years old, on warfarin for venous thromboembolism) and “I have an appointment every 3 weeks. I talk to them quite a bit. They adjust the medicine to help with bleeding issues. They recommend changes in the diet. Suggestions for the warfarin, which is what they’re giving to me.” (male, 71 years old, on warfarin for atrial fibrillation) Two others (22%) reported minimal monitoring and healthcare interactions, reporting, “I haven’t been seen since starting the medication, but they keep changing things around; one says take a pill twice a day and one says once a day.” (female, 72 years old, on apixaban for atrial fibrillation) Two other respondents noted that they themselves preferred not to talk about bleeding with their providers, stating, “I never talk about bleeding with my doctors because it’s never that much. It’s not enough to cause a problem.” (female, 76 years old, on warfarin for venous thromboembolism).

### Social function

Participants reported variable effects of bleeding on their interpersonal relations. For six (67%), bleeding affected their social interactions with family. For instance, one (11%) noted the impact of bleeding on his role in his marriage: “I do almost all the cooking. I love to cook; it’s kind of my hobby. When it comes to chopping and carving, my wife prefers that she do it rather than me.” (male, 86 years old, on warfarin for venous thromboembolism) Two (22%) reported that although bleeding was on their minds, they were still able to interact with family and friends according to their goals, noting, “I am just grateful to do my housekeeping and meals and drive and carpool grandkids and things like that. My life is still good.” (female, 81 years old, on apixaban for atrial fibrillation) Two (22%) noted that they had found community with others affected by bleeding: “I go to visit people who have had similar things. I tell them: ‘Sure would be nice if you had a railing. Just be aware, be cautious. Listen to that voice that tells you and don’t think you know more.’” (female, 86 years old, on apixaban for atrial fibrillation).

### Emotional function

Seven (78%) participants reported that bleeding affected their emotional wellbeing. Five (56%) participants noted a sense of constant mental vigilance that affected mood, noting, “Sometimes it affects my mood when I think, ‘What if the scrape went deeper and damaged a blood vessel?’” (female, 76 years old, on warfarin for venous thromboembolism) Two (22%) expressed that bleeding caused them to be fearful, reporting that, “One time I was scared because it seemed near a blood vessel. I was reaching down near the car console, and I saw my hand, it was bleeding.” (male, 69 years old, on rivaroxaban for venous thromboembolism) Three (33%) others noticed that bleeding had affected their motivation. One said, “I don’t have the ambition that I had. I would identify myself as lazy to tell you the truth, and I’m not getting things done the way I would like to.” (male, 86 years old, on rivaroxaban for venous thromboembolism) Three (33%) reported a sense of vulnerability, “Because of that I’m really careful about walking anywhere. I’m always aware of ‘what if I fall?’ because that can kill you. I worry about that, as far as being mobile.” (male, 69 years old, on rivaroxaban for venous thromboembolism) Two (22%) participants highlighted sources of resiliency, including close family and friend relationships, as well as fulfilling activities not affected by bleeding like knitting and driving.

### Physical function

Seven (77%) participants noted effects on their physical function because of bleeding. For six (67%), bleeding further limited already-reduced exercise: “I don’t do a lot of exercise right now because of worries about bleeding.” (male, 71 years old, on warfarin for atrial fibrillation) This extends beyond exercise to mobility, with one noting, “I had the habit of closing the front door with my shoulder when I come in the house, and I had to stop that because it led to a large bruise just from the pressure putting on it.” (female, 86 years old, on apixaban for atrial fibrillation) Two others (22%), however, were still able to maintain physical activities that brought them joy despite bleeding: “I quit going down the water slide, but I still ride the jet ski.” (female, 81 years old, on apixaban for atrial fibrillation).

### Results of semi-structured qualitative interviews of pilot measure

Semi-structured qualitative interviews informed the revision of items in the pilot measure, which can be found in S1 Table. After three semi-structured qualitative interviews, three items were revised due to lack of clarity. We added dental procedures in addition to medical procedures as potential harbingers of bleeding symptoms. We added walking for exercise as a form of physical activity. We changed resilience to coping with stress or unexpected problems. After an additional three interviews, three items were revised to improve understanding. We removed carrying items as an example of mobility; we deleted reference to the healthcare system; and we changed the word supplements to vitamins. One item was revised due to lack of clarity after the following three interviews: rather than asking about healthcare concerns, we asked about healthcare experiences. Finally, one additional item was revised after the next three interviews: we changed minor wound bleeding to bleeding from cuts or injuries. Item revision was no longer required after this interview. The last seven participants demonstrated an understanding of all items, and no further interviews were conducted.

## Discussion

In this study of racially and ethnically diverse older adults eligible for anticoagulation due to AF or VTE, we found that bleeding had a multifaceted effect on QoL that would not be measured by existing clinical outcomes or previously validated questionnaires [6, 27–31]. In response, this paper reports the first steps in the process of developing a new condition-specific PROM. Using focus groups, we refined a hypothesized conceptual model that contains specific domains that constitute bleeding-related QoL in older adults. After generating a draft measure, data from semi-structured qualitative interviews were used to revise items so that they were understandable, comprehensive and not redundant. This novel measure has been reviewed by experts and is now undergoing psychometric testing, which will ultimately lead to a final measure for bleeding-related QoL for older adults.

Some facets we identified are part of established QoL measures, but several are unique to bleeding in older adults. Within generic QoL measures, our results emphasized that specific subdomains within physical, emotional and social functioning matter more than others, including day-to-day activities, sense of accomplishment, and contribution to family responsibilities [28, 31]. Our results also are distinct from two tools developed specifically to measure treatment satisfaction measures for anticoagulants, the DASS and ACTS [6, 7]. First, these existing tools measure treatment satisfaction, which is a related but different patient-reported outcome. Although most of our participants were taking anticoagulant medications, only one discussed issues with the medication itself, which are an outsize focus of the DASS and ACTS. Finally, bleeding’s effects on emotional and social wellbeing were a more prominent concern for participants in our analysis. These differences support the development of dedicated measures in specific populations to enable patient-centered care.

Although our main purpose was to develop a novel PROM, our results may also have relevance to the clinical care of older adults with bleeding. Participants described bothersome bleeding symptoms that may not be routinely screened for, including lab draws, bleeding from minor wounds, and bruising. Clinicians may need to expand screening questions for bleeding symptoms to include not only traditional large-volume bleeding, such as gastrointestinal bleeding, but covert symptoms like bruising. Moving away from the pejorative terminology “nuisance bleeding” to describe small-volume bleeding that can, in fact, be debilitating might encourage more open discussion. Importantly, bleeding symptoms were described by both patients taking anticoagulants and those who were not, affirming that bleeding in older adults is often multifactorial. Our method of purposeful sampling aimed to capture these additional bleeding risk factors, such as diagnoses and chronologic age, in addition to anticoagulant effect. We recommend collecting this information when using this measure.

Additionally, many participants dismissed their bleeding symptoms as trivial but then went on to describe how bleeding hinders their QoL. Several participants even indicated they were uncomfortable bringing up bleeding symptoms, even when they significantly compromised their wellbeing. Our findings suggest that clinicians may need to explicitly ask about patients’ experience of bleeding and QoL rather than waiting for patients to volunteer this information, including any misconceptions about “expected” side effects and how even fear of bleeding can impact wellbeing.

The results of this study also have potential policy implications. Participants reported substantial variation in contact with healthcare providers. Notably, patients followed by centralized anticoagulation clinics reported regular, helpful discourse about bleeding with providers as compared with those followed in other clinics. Multidisciplinary anticoagulation clinics were developed to oversee management of patients prescribed vitamin K antagonist anticoagulants, which require frequent monitoring and dose adjustment [32]. With the advent of direct oral anticoagulants, which do not require such extensive monitoring, the continued value of anticoagulation clinics has been questioned [33]. Our findings suggest an ongoing role for centralized care to standardize monitoring and to help intervene to mitigate bleeding’s impact on QoL, particularly in an older, frail, multimorbid population.

Most importantly, insights on the patient experience gleaned from this study lay a foundation for using a condition-specific PROM for future research and clinical care. Our results demonstrate that current bleeding definitions used in clinical trials and other research amplify aspects that physicians, researchers and health systems consider important and neglect many dimensions that patients consider meaningful. The findings highlight the need for and are the evidence-based first steps in the development of a more comprehensive PROM that is meaningful to patients. This will ensure that future interventions to improve bleeding-related QoL, including clinical trials of novel care strategies or medications observational research to examine decision-making, are examined in line with patients’ priorities.

### Limitations

We acknowledge several limitations of this research. Due to funding and workforce limitations, the same two investigators conducted and analyzed the data, which could introduce bias. This study was limited to a single center, and for feasibility, we included only English-speaking participants who were able to complete the focus group or interview. We chose to focus initially on older adults with a chart diagnosis of AF or VTE eligible for anticoagulation since this represents a high-risk therapy in a vulnerable population, and it can be directly compared to other thrombosis-related outcomes. Lastly, our study was not designed to quantitatively measure the degree to which bleeding changed QoL. Our future work will evaluate whether our findings are replicated in other populations to ensure representativeness, including non-English speakers and bleeding unrelated to anticoagulants. We are using these results to develop a novel PROM that will enable future quantitative measurement of bleeding-related QoL.

## Conclusion

The experiences of older adults provided insight into the aspects of wellbeing and function affected by bleeding that are meaningful to patients and informed development of a new condition-specific PROM ready for psychometric evaluation.

## Supporting information

S1 File A and B: Focus group guide and Pilot measure interview guide. (DOCX)

S1 Table Item revision for novel patient-reported outcome measure. (DOCX)

S1 Checklist COREQ checklist. (PDF)
