# Supplementary material for: The impact of anticoagulant-related bleeding on quality of life: Development of a novel measure based on perspectives from older adults
Source: PLoS One. 2025 Jan 29;20(1):e0316796. doi: 10.1371/journal.pone.0316796 (PMC11778767; doi:10.1371/journal.pone.0316796)
Supplement: S1 File — (DOCX) [file pone.0316796.s001.docx]

**Supplement 1 for:** The Impact of Anticoagulant-Related Bleeding on Quality of Life: Development of a Novel Measure Based on Perspectives from Older Adults

**S1 A Focus group guide**……………………………………….…………Pages 2-4

**S1 B Interview guide**…………………………………………...………….Pages 5-8

**S1 A: Focus group guide**

**Introduction:**

Good *morning/afternoon* and welcome to our focus group discussion. The purpose of today’s discussion is to hear your thoughts and opinions about the impact of bleeding on your life. We hope that the information we learn from our focus groups will improve the quality of care patients receive. So your view is very important. I want to thank you for taking time out of your day to meet with us because we know that you are very busy, and we really appreciate your contribution to this project.

My name is *________*, and my role today is to serve as the moderator for our group discussion. Assisting me is *_________*.

This discussion will be tape recorded so we don’t miss any of your comments, and we will be taking notes as well. Our focus group discussion will take no more than 1 hour unless you really get talking. We will be asking you questions about the effects of bleeding on your life. No names will be included in any reports. All of your comments will remain confidential.

**Ground Rules:**

There are no right or wrong answers to the questions I am about to ask. We expect that you will have differing points of view. Please feel free to share your point of view even if it is different from what others may have said. If you want to follow up on something someone has said, you want to agree, disagree, or give an example, feel free to do that. I am here to ask questions, listen and make sure everyone has a chance to share. We’re interested in hearing from each of you and we want to make sure everyone has a chance to share their ideas.

**Discussion:**

1. I’d like to start our main topic of discussion today, which is the impact of bleeding on your life. Let’s go around the room and describe any experiences with bleeding you may have had, if any.

*Prompt: If specific bleeding symptoms are not mentioned, then the moderator will specifically ask about these symptoms (e.g. “Other patients have told us that these are important- have any of you experienced these other bleeding symptoms?)*

- - *Nosebleed*
  - *Skin bleeding or bruising*
  - *Minor wound bleeding*
  - *Blood in urine*
  - *Blood in bowel movements*
  - *Mouth bleeding*
  - *Bleeding after procedures*
  - *Vaginal bleeding*
  - *Bleeding into a muscle or joint (usually diagnosed by a physician)*
  - *Other (blood draws, etc.)*

1. Now I want you to think about a typical day for you—going to work, taking care of your family, working around the house—whatever your typical day consists of. What specific bleeding symptoms have bothered you most in your daily life.

*Prompt: Moderator in round-robin fashion asks each participant to state his/her responses.*

Now let’s rank these symptoms from those with highest impact to lowest impact. What makes these symptoms low or high impact?

*Prompt: Moderator then goes round robin and asks participants to rank symptoms from low to high*

What do others think?

Has the impact or amount of bother from these symptoms changed for you over time? Do you have some ways that you’ve learned to cope with these symptoms?

1. We are now switching gears a little. Next, think about specific activities that are affected by bleeding or fear of bleeding. Could someone share any activities you feel bleeding impacts?

*Prompt:*

*Moderator will sort activities into domains as they are discussed.*

*Moderator in round-robin fashion asks each participant to state his/her responses. Next, if specific activities are not mentioned, then the moderator will specifically ask about these activities (e.g. We know from patients that bleeding can affect…).* o *Physical function at home (e.g. yardwork, cleaning)* o *Physical function at work (e.g. manual labor, stairs)* o *Exercise*

- - - *Social function with family (e.g. grandkids)*
    - *Social function with friends, neighbors, groups (e.g. dining out)* o *Mood or affect*
    - *Mental health (anxiety, depression)* o *Energy/vitality*I

1. We’ve written down all of the activities you’ve mentioned. Let’s go through together and rank the activities – activities where bleeding has the greatest impact first, then activities where bleeding has the least impact.

*Prompt: Moderator then goes round robin and asks participants to rank symptoms from low to high*

What do others think?

Has the effect of bleeding on these activities changed over time?

Have you developed ways to deal with the effect of bleeding on these activities?

1. Bearing in mind all of the things we’ve discussed above, when you see your provider, what kind of things do you talk about? Do you talk about the symptoms we discussed above? Do you talk about the impact of bleeding on your daily lives?

1. Thinking about your past visits, tell me about anything you did not get addressed that you would have hoped to? What do you think is not important for them to ask about?

1. We are coming to the end of our discussion. Is there anything that we missed or that you came wanting to say that you didn’t get a chance to say about life as a person at risk of bleeding? Please let us know if you’d like to add anything.

**Conclusion:**

Great! Thank you all so much for participating in our focus group. This has been a wonderful discussion. Your opinions will be a valuable asset to the improvement of care for persons at risk for bleeding. We hope you have found the discussion interesting! I would also like to remind you that any comments featured in our report will be anonymous. I will give you your gift card. We would love to include you in future parts of the study, so we will contact you once these pieces are open: one is more in-depth one-on-one interviews, and then we would also love for you to take the final questionnaire we are developing.

**S1 B: Semi-structured qualitative interview guide**

**Introduction:**

Good *morning/afternoon* and thank you for joining us for this interview. The purpose of today’s interview is to hear your thoughts and opinions on a new questionnaire we are developing about the impact of bleeding on your life. We hope that the information we learn from you will improve the quality of care patients receive. So your view is very important. I want to thank you for taking time out of your day to meet with us because we know that you are very busy, and we really appreciate your contribution to this project.

My name is *________*, and my role today is to serve as the interviewer. Assisting me is *_________*.

This interview will be tape recorded so we don’t miss any of your comments, and we will be taking notes as well. Our interview will take no more than 1 hour unless you really get talking. We will be asking you questions about bleeding on your life. No names will be included in any reports. All of your comments will remain confidential.

**Ground Rules:**

There are no right or wrong answers to the questions I am about to ask. Please feel free to share your point of view. You are free to stop the interview at any point or skip any question at any point. I am here to ask questions, listen and make sure you have a chance to share. We’re interested in hearing about your experiences.

**Draft Questionnaire**

**Bleeding symptoms**

**During the last 4 weeks, how often have you experienced the following symptoms (circle one answer on each line)?**

(1 = never, 2 = less than once a month, 3 = about once a month, 4 = once a week, 5 = every day).

1. Nosebleeds

1. Skin bleeding or bruising

1. Minor wound bleeding

1. Bleeding in your bowel movements

1. Bleeding after medical or dental(?) procedures

1. Other bleeding (e.g. in urine, coughing up blood, vaginal, etc…)

**Physical function**

**How much does bleeding (or the possibility of bleeding) affect your ability to participate in the following physical activities (circle one answer on each line)?** (1 = not at all, 2 = a little, 3 = moderately, 4 = quite a bit, 5 = extremely)

1. Daily activities at home (e.g. housework, ironing, doing odd jobs/repairs around the house, gardening, etc.…)

1. Mobility (e.g. walking, climbing stairs, getting into and out of a car, carrying items, etc…)

1. Exercise (e.g. biking, swimming, running, hiking, skiing, etc…)

**Emotional function**

**How much does bleeding (or the possibility of bleeding) affect these aspects of your emotional health (circle one answer on each line)?**

(1 = not at all, 2 = a little, 3 = moderately, 4 = quite a bit, 5 = extremely)

1. Mood

1. Energy level

1. Resilience

**Social function**

**How much does bleeding (or the possibility of bleeding) affect your ability to participate in the following social activities (circle one answer on each line)?** (1 = not at all, 2 = a little, 3 = moderately, 4 = quite a bit, 5 = extremely)

1. Interactions with family

1. Interactions with friends

1. Interactions with other groups (e.g. work, volunteering, neighbors, church, etc.)

1. Travel

**Healthcare experiences**

**How much does bleeding (or the possibility of bleeding) affect the following other health concerns?**

(1 = not at all, 2 = a little, 3 = moderately, 4 = quite a bit, 5 = extremely)

1. Other medications (e.g. aspirin, blood pressure medication, supplements, etc…)

1. Other health conditions or symptoms (e.g. heart disease, high blood pressure, diabetes, falls, etc…)

1. Interactions with healthcare providers or the healthcare system (e.g. nurses, doctors,

clinic staff, etc…)

## Interview questions

## Comprehension

- How was it to read and understand the questions?
- Prompt: Tell me more about that
- Were there any questions you had to read more than once?
- Prompt: Which questions and why?
- Were there any questions you had to think more about?
- Prompt: Which questions and why?

## Instructions

- How was it to read and understand the instructions?
- Prompt: Tell me more about that.
- Were there any instructions you had to read more than once?
- Prompt: Which instructions and why?
- How would you improve the instructions?
- Prompt: Why? Tell me more about that.

## Clarity

- Are there any items that could be misunderstood?
- Prompt: How would you rephrase it?
- Were there any words that you think could be misunderstood?
- Prompt: How would you rephrase it?

## Relevance

- Were there any questions you felt didn’t belong, were out of place or seemed not relevant? o Prompt: Can you tell me why?
- Were there any topics you felt like weren’t covered?

o Prompt: What questions would you add?

## Sensitivity/Bias

- Were there any questions that felt too sensitive to answer?
- Prompt: Why? How would you have asked?
- Is there any wording you would change?
- Prompt: Why?

## Structure

- How was the order of the questions?
- Prompt: Tell me more.
- Prompt: Were there any questions you missed?
- Prompt: Are there any questions or instructions you would move and why?

## Other

• Were there any other problems with the questions or the instructions that I haven’t asked about?
