# Supplementary material for: The impact of anticoagulant-related bleeding on quality of life: Development of a novel measure based on perspectives from older adults
Source: PLoS One. 2025 Jan 29;20(1):e0316796. doi: 10.1371/journal.pone.0316796 (PMC11778767; doi:10.1371/journal.pone.0316796)
Supplement: S1 Table — (DOCX) [file pone.0316796.s002.docx]

**Supplement 2 for:** The Impact of Anticoagulant-Related Bleeding on Quality of Life: Development of a Novel Measure Based on Perspectives from Older Adults

**S2 A Table Item revision for novel patient-reported outcome measure**……….…Pages 2-4

**Table: Item revision for novel patient-reported outcome measure**

| Domain | Theme | Item | Important observations | Outcome |
| --- | --- | --- | --- | --- |
| Bleeding symptoms | Bleeding after procedures | **During the last 4 weeks, how often have you experienced bleeding after medical procedures?** | Bleeding after dental procedures (teeth cleaning, root canal) mentioned repeatedly | Added dental procedures to item. |
| Physical function | Exercise | **How much does bleeding (or the possibility of bleeding) affect your ability to exercise (e.g. biking, swimming, running, hiking, skiing, etc…)** | Walking discussed in mobility, but several participants described walking solely for exercise | Added walking for exercise to item. |
| Emotional function | Resilience | **How much does bleeding (or the possibility of bleeding) affect your resilience?** | All participants asked for this question to be repeated or clarified. | Changed resilience to coping with stress or unexpected problems (defined resilience) |
| Physical function | Mobility | **How much does bleeding (or the possibility of bleeding) affect your mobility (e.g. walking, climbing stairs, getting into and out of a car, carrying items, etc…)?** | When asked if carrying items was relevant, all participants said it was not | Removed carrying items from item. |
| Healthcare experiences | Interactions with healthcare providers or system | **How much does bleeding (or the possibility of bleeding) affect your interactions with healthcare providers or the healthcare system (e.g., nurses, doctors, clinic staff, etc…)** | Participants had difficulty the meaning of healthcare system. | Removed healthcare system from item. |
| Healthcare experiences | Other medications | **How much does bleeding (or the possibility of bleeding) affect your other medications (e.g. aspirin, blood pressure medication, supplements, etc…)** | When queried, participants had greater understanding of vitamins rather than supplements | Changed supplements to vitamins. |
| Healthcare experiences | Healthcare experiences | **How much does bleeding (or the possibility of bleeding) affect the following other health concerns?** | Participants had a negative emotional reaction to “concerns” and preferred “experiences” | Changed concerns to experiences |
| Bleeding symptoms | Minor wound bleeding | **During the last 4 weeks, how often have you experienced minor wound bleeding?** | Participants suggestion clarification of what wound meant | Changed wound to bleeding from cuts or injuries |
